# Notes and News

**Published:** 1897-12-04

**Authors:** 


### NOTES AND NEWS.

(Collected and Communicated.)

MEETINGS.

A meeting of the Röntgen Society will be held at the Medical Society's Rooms, 11, Chandos Street, on Tuesday, December 7th, at 8 30, when a paper by Mr. A. Campbell Swinton will be read on "Adjustable X-Ray Tubes."

The annual meeting of the Surgical Aid Society will be held at the Mansion House, on Tuesday, December 7th, at 3 p.m., the Lord Mayor presiding.

The total amount received by the Prince of Wales's Hospital Fund now amounts to £188,751, irrespective of the sum resulting from the sale of the Hospital Stamps.

We regret to learn that Mr. Newstead, secretary of the Royal London Ophthalmic Hospital, has retired from his post through ill-health, from which he has been suffering for some time.

At the last meeting of the Council of the Metropolitan Hospital Sunday Fund, the annual meeting of the Fund was fixed for December 13th, when it will be recommended that June 12th shall be appointed Hospital Sunday in 1898.

Mr. Rudyard Kipling, who was the guest of the evening at the complimentary dinner to Sir William Gowers given by the Society of Medical Phonographers, spoke in eulogistic terms of the courageous devotion to duty exhibited by medical men, of which he himself had witnessed striking examples.

Fifty beds are to be closed at University College Hospital, the funds of the institution not being equal to maintaining them. We regret to learn that such a course has been found necessary, for this hospital serves a poor and crowded district, and its claims should be powerful enough to secure most adequate support, even within its own vicinity.

We are glad to see that the *Lancet* has taken up the cudgels on behalf of the horses who suffer so terribly from the greasy condition of London streets during the major portion of the year. The evil of dirty streets is so universally accepted as a matter of course in London, that it is to be feared that great pressure will have to be brought to bear, and persistent effort resorted to before any reform will take place.

Amongst the new list of Mayors throughout the country the names of eleven medical men are to be found.

The secretaries of the Prince of Wales's Hospital Fund have received £362 from the Lady Mayoress of the past year, making a sum of £5,362 in all secured by her exertions for the Fund.

An old trick has recently again been played upon charitable institutions. A letter is sent to the secretary of a hospital or some other institution by a writer who expresses himself desirous to bequeath money to the charity, and who asks for an introduction to a reliable London solicitor or stockbroker. Armed with the introduction obtained, he is able to approach the firm as a client for his own purposes.

A curious land dispute has just been in the law courts in connection with the Lincoln County Hospital. A late governor of the institution, possessing land adjacent to it, had entered into an arrangement not to build within 20 yards of the hospital. On his death a firm of builders, who acquired the land, commenced building within the vetoed limit. The hospital authorities applied for an injunction against the builders, which they succeeded in obtaining.

The festivities connected with the opening of the new General Hospital at Birmingham still continue, and illustrate the immense amount of public interest that hospitals excite in the present day. The most recent deed of commemoration of the establishment of the Birmingham Hospital was a dinner given by the committee to entertain Mr. Holder, and to present him with an illuminated address as a memento of the completion of the magnificent institution. As chairman of the Building Committee, and previously during his connection with the hospital, Mr. Holder has exhibited the most lively interest, energy, and generosity on its behalf.

The Huntingdon County Hospital has lately been thoroughly renovated and so far enlarged as to accommodate 42 patients. Mr. Keith D. Young was consulted by the board of management, and his suggestions have been carried out, under the direction of Mr. E. Borisson, architect, of Huntingdon. Messrs. Roberts, of Islington, have laid down teak floors in all the wards; and Messrs. Dent and Hellyer, of Newcastle Street, have furnished the newest sanitary appliances. New bedsteads have been supplied by the Longford Wire Company, and each ward has one of Dr. Pridgin Teale's fireplaces. A new little operating theatre has been built at the cost of one of the governors; it is floored with terrazzo, and lined throughout with opalite. Two kinds of lockers have been adopted in the wards, some by Messrs. Shoolbred, similar to those in use at the Poplar Hospital: others of iron and glass, of the Berlin pattern at the Evelina, have been made by Messrs. Ponders and Baker, of Clerkenwell Road. A mortuary chapel, the gift of another governor, is being built, and will be decorated by Messrs. Powell, of Whitefriars. The hospital is well situated on a breezy common near the Huntingdon Great Northern Railway Station.

